# Genomic highlights of the phylogenetically unique halophilic purple nonsulfur bacterium, *Rhodothalassium salexigens*

**DOI:** 10.1007/s00792-025-01380-8

**Published:** 2025-01-25

**Authors:** Michael T. Madigan, Kelly S. Bender, Mary N. Parenteau, Yukihiro Kimura, Zheng-Yu Wang-Otomo, W. Matthew Sattley

**Affiliations:** 1 School of Biological Sciences, Program in Microbiology, Southern Illinois University, Carbondale, Illinois USA; 2 Exobiology Branch, NASA Ames Research Center, Moffett Field, California USA; 3 Department of Agrobioscience, Graduate School of Agriculture, Kobe University, Nada Kobe, Japan; 4 Faculty of Science, Ibaraki University, Mito, Japan; 5 Division of Natural Sciences, Indiana Wesleyan University, Marion, Indiana USA

**Keywords:** Halophile, Purple nonsulfur bacteria, *Rhodothalassium salexigens*, Genome

## Abstract

*Rhodothalassium* (*Rts.*) *salexigens* is a halophilic purple nonsulfur bacterium and the sole species in the genus *Rhodothalassium*, which is itself the sole genus in the family *Rhodothalassiaceae* and sole family in the order *Rhodothalassiales* (class *Alphaproteobacteria*). The genome of this phylogenetically unique phototroph comprises 3.35 Mb and is highly chimeric, with nearly half of its genes originating from families other than the *Rhodothalassiaceae*, many of which lack phototrophic species. Photosynthesis genes in *Rts. salexigens* are not arranged in a typical photosynthesis gene cluster but are scattered across the genome, suggesting an origin from horizontal transfers. Despite an encoded RuBisCO, autotrophy has not been observed in *Rts. salexigens*, and enzymes that oxidize common inorganic electron donors are not encoded. Phospholipid biosynthesis in *Rts. salexigens* is restricted, and phosphoglycerolipids are the only phospholipids present in its intracytoplasmic membranes. *Rts. salexigens* fixes nitrogen using a Mo-containing nitrogenase and uses ammonia despite previous results that indicated it was a glutamate auxotroph. Glycine betaine is the sole osmolyte in *Rts. salexigens*, and enzymes are encoded that facilitate both its uptake and its biosynthesis from glycine. The genomic data also support chemotactic swimming motility, growth over a range of salinities, and the production of membrane-strengthening hopanoids.

## Introduction

Several phototrophic purple bacteria are halophilic, and although most grow best near marine salinities (3–4%), a few species of purple sulfur (PS) bacteria (*Gammaproteobacteria*) are among the most halophilic of all known *Bacteria*, growing optimally near 15–20% NaCl and slowly in salt-saturated culture media. By contrast to halophilic PS bacteria, halophilic purple nonsulfur bacteria (PNS) are *Alphaproteobacteria*, and most grow optimally at marine salinities. However, three species can grow in media containing 20% NaCl; these include two *Rhodovibrio* (*Rhv*.) species—*Rhv. salinarum* and *Rhv. sodomensis*—and *Rhodothalassium* (*Rts*.) *salexigens*. *Rhv. salinarum* grows optimally at 4% NaCl (Imhoff 2001), whereas *Rhv. sodomensis* grows optimally at 12% NaCl (Mack et al. 1993). By contrast to both of these, *Rts. salexigens* displays a salinity optimum of 7% (Drews 1981; Borghese et al. 2001; Imhoff 2001; Imhoff et al. 2019), a value above that of marine PNS bacteria but below that of *Rhv. sodomensis*. Some phenotypic properties of these three halophilic PNS bacteria are summarized in Table 1.

**Table 1:** Some phenotypic properties of *Rhodothalassium salexigens*, *Rhodovibrio salinarum*, and *Rhodovibrio sodomensis*^a^

Characteristic	*Rts. salexigens*	*Rhv. salinarum*	*Rhv. sodomensis*
Type strain	WS-68 (DSM 2132)	DSM 9154	DSI (DSM 9895)
Habitat	Seawater evaporation ponds	Solar salterns	Dead Sea
Salinity optimum (range)	7% (5–20)	4% (3–24)	12% (6–20)
Glycine betaine as osmolyte	Yes	Yes	Yes
Choline betaine as osmolyte	No	Yes	Yes
Ectoine as osmolyte	No	Yes	Yes
Photocomplexes	LH1^b^, LH2	LH1, LH2	LH1
Absorption maxima (nm)	800, 840, 875^c^	802, 873	875
ICM^d^	Concentric layers	Vesicular	Vesicular

^a^All species are *Alphaproteobacteri*a (see Fig. 1) and contain bacteriochlorophyll *a* and spirilloxanthin (or spirilloxanthin derivatives) as major carotenoids. Data from Drews (1981), Mack et al. (1993), and Nissen and Dundas (1984)

^b^*LH* light-harvesting

^c^See absorption spectrum in Fig. 4

^d^*ICM* intracytoplasmic photosynthetic membranes

**Fig. 1:**
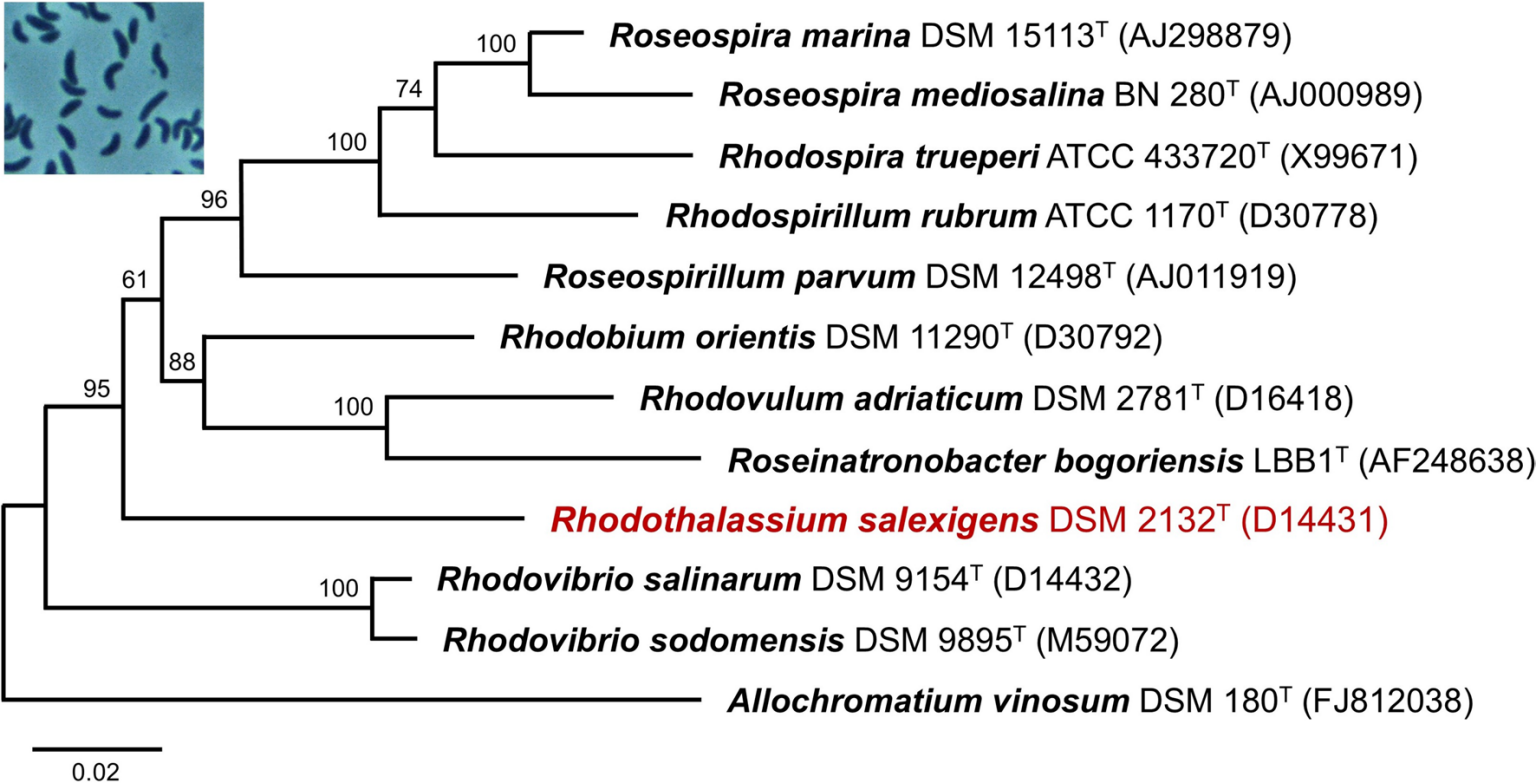
Phylogeny of *Rts. salexigens*. Neighbor-joining phylogenetic tree of several purple nonsulfur bacteria in the *Alphaproteobacteria* based on comparative 16S rRNA gene sequences (1379 nucleotide positions) using the maximum likelihood method. All species except for *Rsp. rubrum* and *Alc. vinosum* are halophiles. *Alc. vinosum* (*Gammaproteobacteria*) is the outgroup. The scale bar indicates the number of base substitutions per site, and bootstrap values > 60 (based on 1000 replicates) are indicated at the nodes. Genbank accession numbers are shown in parentheses. Inset: Phase-contrast photomicrograph of phototrophically grown cells of *Rts. salexigens*

*Rts. salexigens* strain WS 68 was isolated by Professor William Sistrom from a pond of evaporating seawater on the coast of Oregon (USA) and was later described as a new species of the PNS genus *Rhodospirillum* (*Rsp*.) as *Rsp. salexigens* (Drews 1981). In a taxonomic rearrangement of spiral-shaped purple nonsulfur bacteria (Imhoff et al. 1998), the genus name of strain WS 68 was changed to *Rhodothalassium* while the species epithet, *salexigens*, was retained. *Rts. salexigens* is phylogenetically unique, with the sole species in the genus forming its own family- and order-level taxa (Ramana et al. 2013). *Rts. salexigens* has no especially close relatives and branches on a 16S ribosomal RNA gene tree between a clade that defines the other extremely halophilic PNS bacteria described in Table 1 and a large clade of marine PNS bacteria (Fig. 1). Cells of all the extremely halophilic species are vibrio- to spirillum-shaped, including those of *Rts. salexigens* (Fig. 1 inset).

Because of its phylogenetic distance from other PNS bacteria, its unusual halophilicity, and several metabolic anomalies noted in various studies, we generated a draft sequence of the *Rts. salexigens* genome and describe here some highlights of this phototroph’s gene complement. Our results help to paint a more comprehensive picture of the biology of *Rts. salexigens* and provide a genetic rationale for some of the unusual physiological features thus far observed in this halophilic PNS bacterium.

## Materials and methods

A culture of *Rhodothalassium salexigens* (basonym *Rhodospirillum salexigens*) strain WS 68 (DSM 2132^ T^) was obtained by MTM from Professor Gerhard Drews, Universität Frieburg, Germany in 1983. Cells were grown phototrophically in the medium described by Rubin and Madigan (1986), and aliquots of late exponential phase cultures were supplemented with 10% glycerol and frozen at –80 °C until thawed and revived as growing cultures for genomic isolation and analysis in this study.

Genomic DNA from a cell pellet of *Rts. salexigens* was isolated using the JetFlex^™^ Genomic DNA Purification Kit (ThermoFisher). Whole-genome shotgun sequencing was done using Illumina NovaSeq (libraries of 300 bp) and the sequences assembled and annotated by the U.S. Department of Energy Joint Genome Institute (DOE/JGI) as previously described (Madigan et al. 2023). The *Rts. salexigens* strain DSM2132^T^ genome sequence is available in the JGI/IMG database as genome ID 2838098893 and also in Genbank as GCA_004341375.1. A *Rts. salexigens* genome sequence from an independent sequencing study is also accessioned in Genbank as GCF_016583875.1.

Phylogenetic analysis of 16S ribosomal RNA genes was done using MEGA version 11 (Tamura et al. 2021). The *Rts. salexigens* 16S rRNA gene sequence was ClustalW aligned with corresponding sequences from select *Alphaproteobacteria* using *Allochromatium vinosum* (NR074584, *Gammaproteobacteria*) as an outgroup to root the phylogenetic tree. The tree was drawn using MEGA 11 according to the parameters described in the legend to Fig. 1. Average nucleotide identities (ANI) of whole genomes (Table 1) were computed using the JGI/IMG ANI algorithm. A whole-genome tree was generated from a total of 59,083 amino acids (aligned with MUSCLE; Edgar 2004) and 177,249 nucleotides (aligned with the Codon_align function of Biopython; Cock et al. 2009) using “Rapid bootstrapping” (500 rounds) in RAxML (Stamatakis 2014). Concatenated sequences of photosynthetic light-harvesting and reaction center proteins were generated manually, aligned using MUSCLE, and analyzed using MEGA 11. A total of 1605 amino acid positions were used in the final dataset, and evolutionary distances were computed using the Poisson correction method (Zuckerkandl and Pauling 1965). The phylogenetic distribution of genes in *Rts. salexigens* showing 30% or greater sequence identity to genes in the IMG/NCBI databases was generated using Krona (Ondov et al. 2011).

For absorption spectra, intracytoplasmic membranes and purified LH1–RC complexes from *Rts. salexigens* were prepared as described by Tani et al. (2022) and absorption spectroscopy performed on a V-730BIO spectrophotometer (Jasco, Japan). Phospholipids in *Rts. salexigens* were determined by ^31^P-NMR as previously described (Satoh et al. 2023). Photomicrographs were taken as described in Hayward et al. (2021).

## Results and discussion

### Genome characteristics

**Fig. 2:**
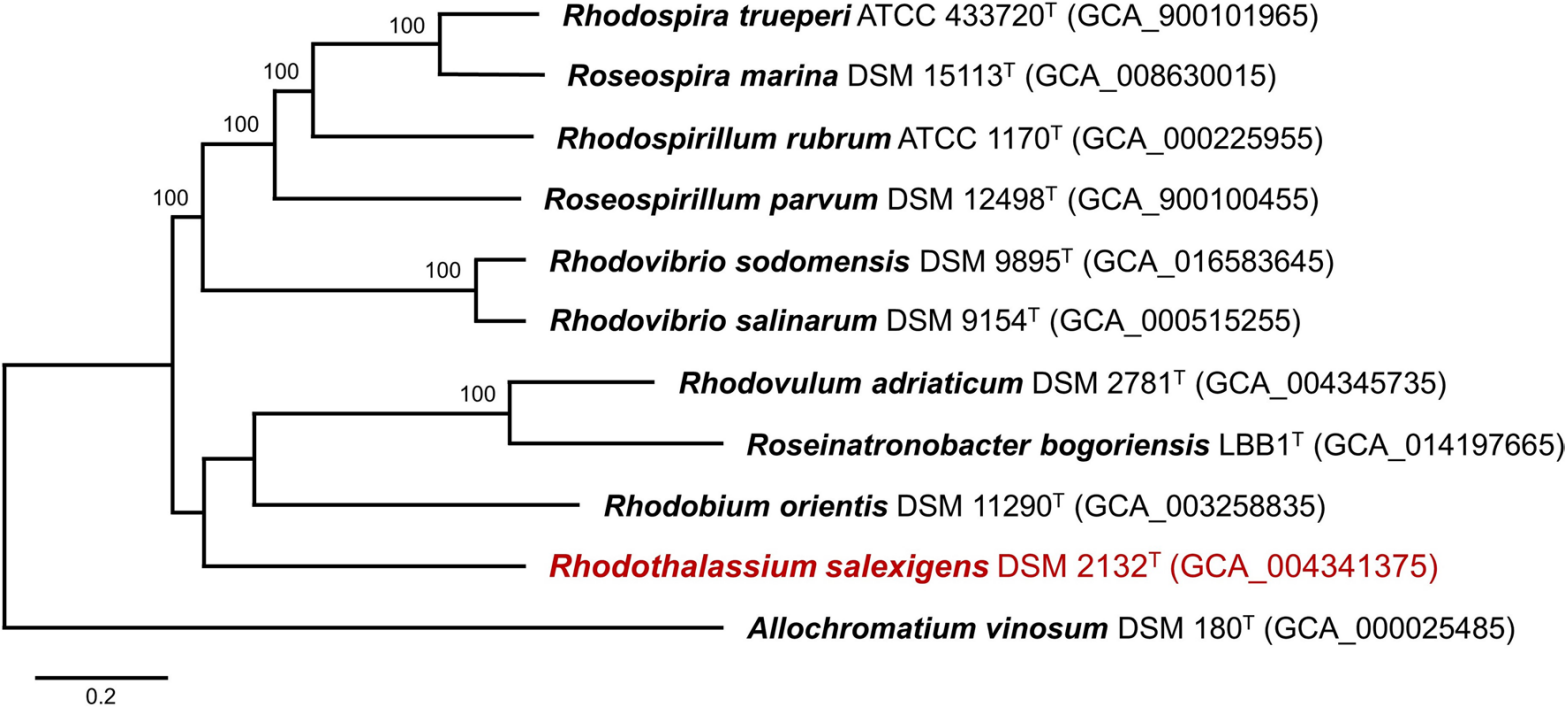
Whole genome phylogenetic tree of the species in Fig. 1 except for *Roseospira mediosalina*, for which a genome sequence is unavailable. The scale bar represents the mean number of substitutions per site averaged across both nucleotide and amino acid changes and bootstrap values are shown at the nodes. Genbank accession numbers are shown in parentheses

The genome of *Rts. salexigens* consists of a circular chromosome of 3,357,861 base pairs; one 18.2 kb plasmid was predicted but its DNA was fragmented and thus this element could not be confirmed. The relatively small size of the *Rts. salexigens* genome contrasts with the significantly larger genomes of the two *Rhodovibrio* species (Table 2). Assembly of the *Rts. salexigens* draft genome sequence yielded 24 scaffolds that contained a total of 2900 protein-encoding genes. Of these, 77.3% were encoded proteins with predicted functions. In agreement with the picture that emerged from 16S rRNA gene sequencing (Fig. 1), comparative sequence analysis of entire genomes confirmed the unique phylogeny of *Rts. salexigens*; the close relationship between the two *Rhodovibrio* species revealed in the 16S rRNA tree was also confirmed in the full genome tree (Fig. 2). Some comparative genomic statistics of these three halophilic PNS bacteria are listed in Table 2.

**Table 2:** Some genomic properties of *Rhodothalassium salexigens*, *Rhodovibrio salinarum*, and *Rhodovibrio sodomensis*

Property	*Rts. salexigens*	*Rhv. salinarum*	*Rhv. sodomensis*	
Genome (Mb)^a^	3.35	4.15	5.75	
Protein-coding genes (% of total)	2900 (97.6)	3806 (98.5)	5730 (97.8)	
G + C content (%)	68.6	65.9	68.4	
ANI (%)^b^	100	73.8	74.0	
tRNA genes	48	48	74	
rRNA genes	4	5	5	
CRISPR arrays	3	1	5	
Calvin cycle genes^c^	Yes	Yes	Yes	
Nitrogen fixation genes^d^	Yes	Yes	No	

^a^Genome IDs (all DOE/IMG): *Rts. salexigens*, 2838098893; *Rhv. salinarum*, 2516653055; *Rhv. sodomensis*, 2993873675

^b^Percent average nucleotide identity to the genome of *Rts. salexigens* (JGI/IMG calculation). ANI between *Rhv. salinarum* and *Rhv. sodomensis* is 85.1%

^c^Autotrophic growth has not been demonstrated in any of the species listed

^d^In *Rts. salexigens*, growth on N_2_ as N source has been confirmed (Rubin and Madigan 1986) but diazotrophic growth of *Rhv. salinarum* has not been demonstrated. Homologs of *nifHDK* are present in the genomes of *Rts. salexigens* and *Rhv. salinarum* but not that of *Rhv. sodomensis*

**Fig. 3:**
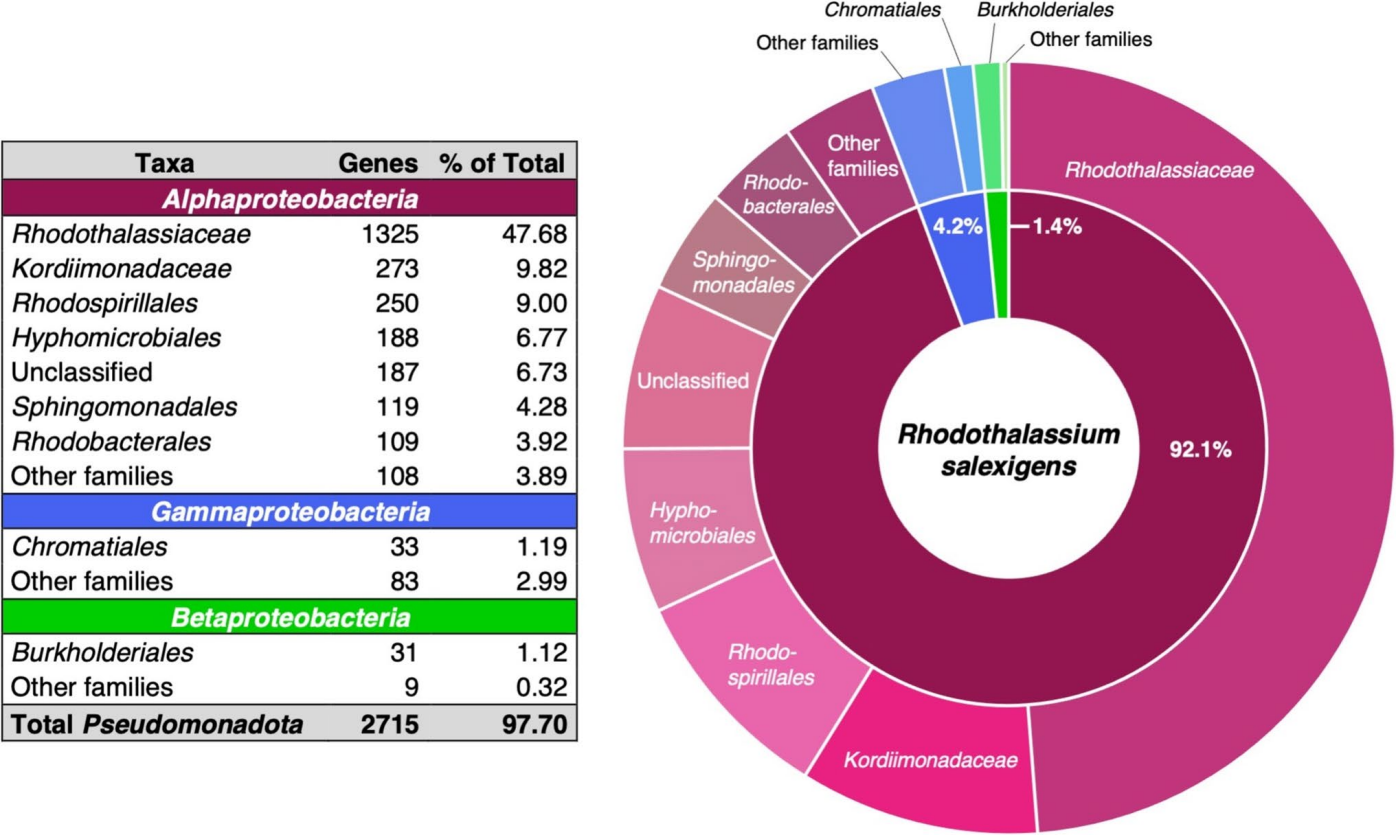
Origins of protein-encoding genes within the phylum *Pseudomonadota* having 30% or higher sequence identity to genes in *Rts. salexigens*. Over half of the genes in the *Rts. salexigens* genome originate from bacterial families outside of the *Rhodothalassiaceae*. The total number of protein-encoding genes in *Rts. salexigens* is 2900. Data extracted from DOE/IMG genome ID 2838098893

Sequence comparisons of protein-encoding genes of *Rts. salexigens* showed its genome to be significantly chimeric. Although more than 90% of the genes in *Rts. salexigens* show > 30% sequence identity to genes from other *Alphaproteobacteria*, virtually half of these genes have their roots in alphaproteobacterial families and orders other than *Rhodothalassiales*, many of which lack known phototrophic representatives (Fig. 3); less than 6% of *Rts. salexigens* genes originate from *Betaproteobacteria* or *Gammaproteobacteria* (Fig. 3). The other half of the *Rts. salexigens* genome has roots within the family *Rhodothalassiaceae*. However, since *Rts. salexigens* is the only known genus in this family, these genes are either unique to this species or to this species and any new *Rhodothlassiaceae* that may be discovered in the future. Few *Rts. salexigens* genes originate from within the *Rhodospirillaceae* or *Paracoccaceae*—families that include well-known PNS bacteria such as *Rhodospirillum* and *Rhodobacter*—or from within the *Ectothiorhodospiraceae*, a family that includes the halophilic PS bacteria *Ectothiorhodospira* and *Thiorhodospira*. By contrast to *Rts. salexigens*, the genome of *Rsp*. *rubrum* is almost exclusively composed of genes with roots in the *Rhodospirillaceae* (Krona analysis of DOE/IMG genome ID637000241). Although not conclusive, the data of Fig. 3 suggest that a large portion of the *Rts. salexigens* genome has arisen from horizontal gene transfers from chemotrophic *Alphaproteobacteria* and with only minimal inputs from other phototrophic purple bacteria, perhaps as few as those genes needed to perform photosynthesis, to be considered now.

### Phototrophy

**Fig. 4:**
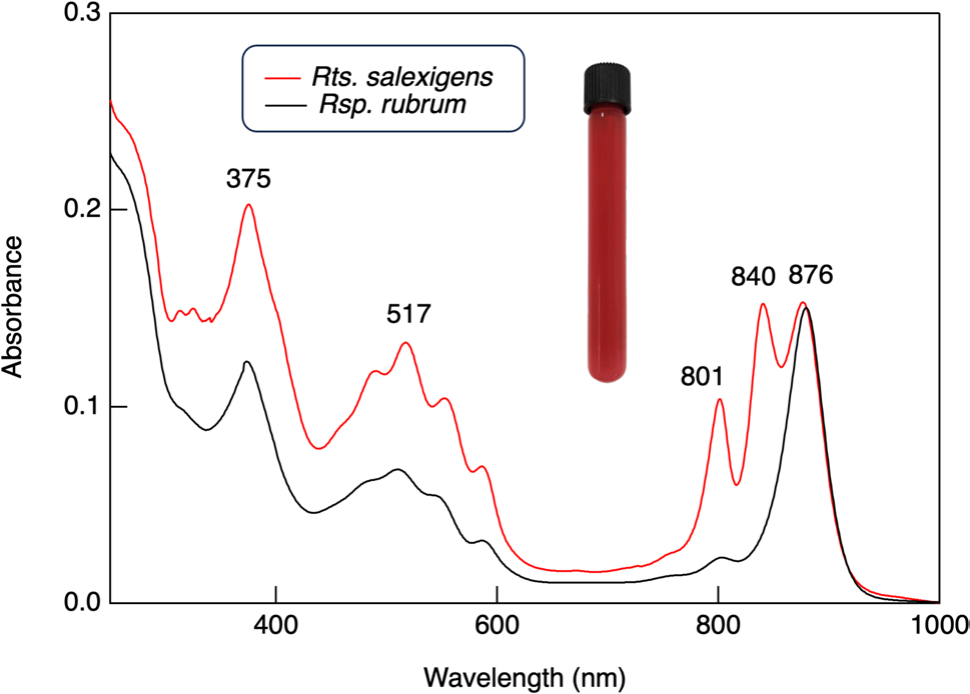
Absorption spectra of intracytoplasmic membranes of *Rts. salexigens* compared with those from *Rsp. rubrum*. Inset: tube culture of phototrophically grown cells of *Rts. salexigens*

*Rts. salexigens* grows chemotrophically (aerobic/dark) by respiration and photoheterotrophically (anoxic/light) using a variety of organic compounds as carbon sources (Drews 1981). Such metabolic flexibility should offer the organism ample growth opportunities in its organic-rich tide pool habitat where both oxic and anoxic regions likely exist. Dense cultures of photoheterotrophically grown cells of *Rts. salexigens* are deep red in color (Fig. 4 inset), and the organism shows major absorbance maxima in the near infrared at 801, 840, and 876 nm. These peaks signal the presence of bacteriochlorophyll (Bchl) *a* and light-harvesting (LH)1 and LH2 photocomplexes (Table 1 and Fig. 4). *Rts. salexigens* produces carotenoids of the normal spirilloxanthin series with spirilloxanthin itself constituting nearly 90% of the carotenoid total (Drews 1981). Levels of carotenoids are notably higher in cells of *Rts. salexigens* than *Rsp. rubrum* (Fig. 4), but much of this could be due to the presence of LH2 in the former and its absence in the latter.

All genes encoding enzymes of the Bchl *a* biosynthetic pathway were identified in the *Rts. salexigens* genome including those encoding key enzymes for the terminal biosynthetic steps through protochlorophyllide *a*, chlorophyllide *a*, and bacteriochlorophyllide *a* to yield phytol-esterified Bchl *a*. Likewise, genes encoding all enzymes necessary to biosynthesize spirilloxanthin from geranylgeranyl-PP through phytoene and lycopene (CrtBI) to spirilloxanthin (CrtCDF) were identified. Small amounts (2–4%) of the total carotenoid pool in *Rts. salexigens* consisted of the intermediates hydroxyspirilloxanthin and rhodovibrin (Drews 1981; Ramana et al. 2013). Besides leading to spirilloxanthin, CrtCDF can also convert neurosporene, a precursor of lycopene, into spheroidene; however, spheroidene pathway carotenoids were not reported in pigment analyses of *Rts. salexigens* (Drews 1981).

**Fig. 5:**
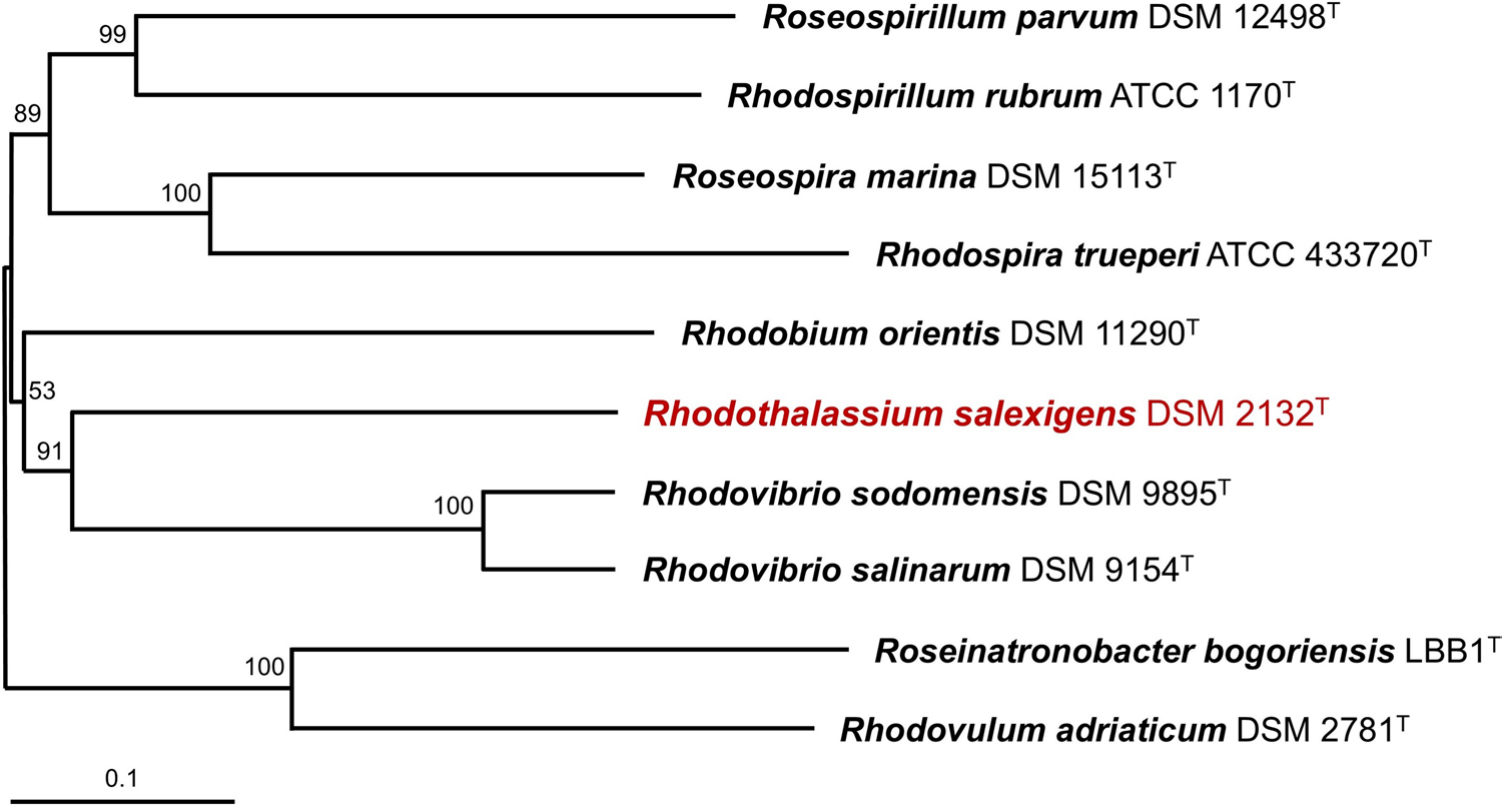
Phylogenetic tree of combined concatenated sequences of the photosynthetic light-harvesting and reaction center proteins PufBALM and PuhABC from the purple bacteria shown in Figs. 1 and 2. Bootstrap values (1000 replicates) are shown at the nodes, and the scale bar represents the number of amino acid substitutions per site

*Rts. salexigens* produces both an LH1–RC core photocomplex and an LH2 (peripheral) antenna complex (Drews 1981; Wacker et al. 1988). Genes encoding reaction center proteins PufML and PuhABC were identified in the *Rts.* s*alexigens* genome, as were the genes *pufAB*, which encode the LH1 alpha and beta polypeptides, respectively. A gene encoding PufC, a cytochrome subunit present in the reaction center of some purple bacteria, was also present in the *Rts. salexigens* genome. Paralleling the 16S rRNA gene and whole genome phylogenies (Figs. 1 and 2), comparative sequencing of a concatenated protein consisting of PufBALMPuhABC, a protein that includes both LH1 and all reaction center proteins, also supports the phylogenetic position of *Rts. salexigens* as being distinct from all other purple bacteria but most closely related to *Rhodovibrio* species (Fig. 5). A similar conclusion was reached from a phylogenetic study of the reaction center and bacteriochlorophyll biosynthesis proteins PufHLM and BchXYZ from a large group of phototrophic *Pseudomonadota* including *Rts. salexigens* (Imhoff et al. 2019). Collectively, then, genes encoding 16S rRNA (Fig. 1), full genome comparisons (Fig. 2), and genes encoding key proteins of photosynthesis (Fig. 5; Imhoff et al. 2019) all point to the unique phylogenetic status of *Rts. salexigens*.

Confirming spectral evidence of an LH2 component in *Rts. salexigens* (Fig. 4), *pucAB*, which encode the alpha and beta subunits of the B800-850 complex, were identified in the genome, as was a homolog of *pucC*, a protein that assists in the assembly of light-harvesting complexes. Homologs of *puhBCE*, which encode photosynthetic complex assembly proteins in the PNS bacterium *Rhodobacter capsulatus* (Aklujar et al. 2005), were annotated as putative assembly proteins in the *Rts. salexigens* genome, with sequence identities near 30%. As expected, a homolog of *pufX*, a polypeptide crucial for dimerization of LH1–RC in *Rhodobacter* species (Tani et al. 2022), was absent from the *Rts. salexigens* genome, as was *pufQ*, a gene encoding a regulatory protein that functions as a metabolic switch between photosynthesis and respiration in most *Rhodobacter* species (Chidgey et al. 2017).

**Fig. 6:**
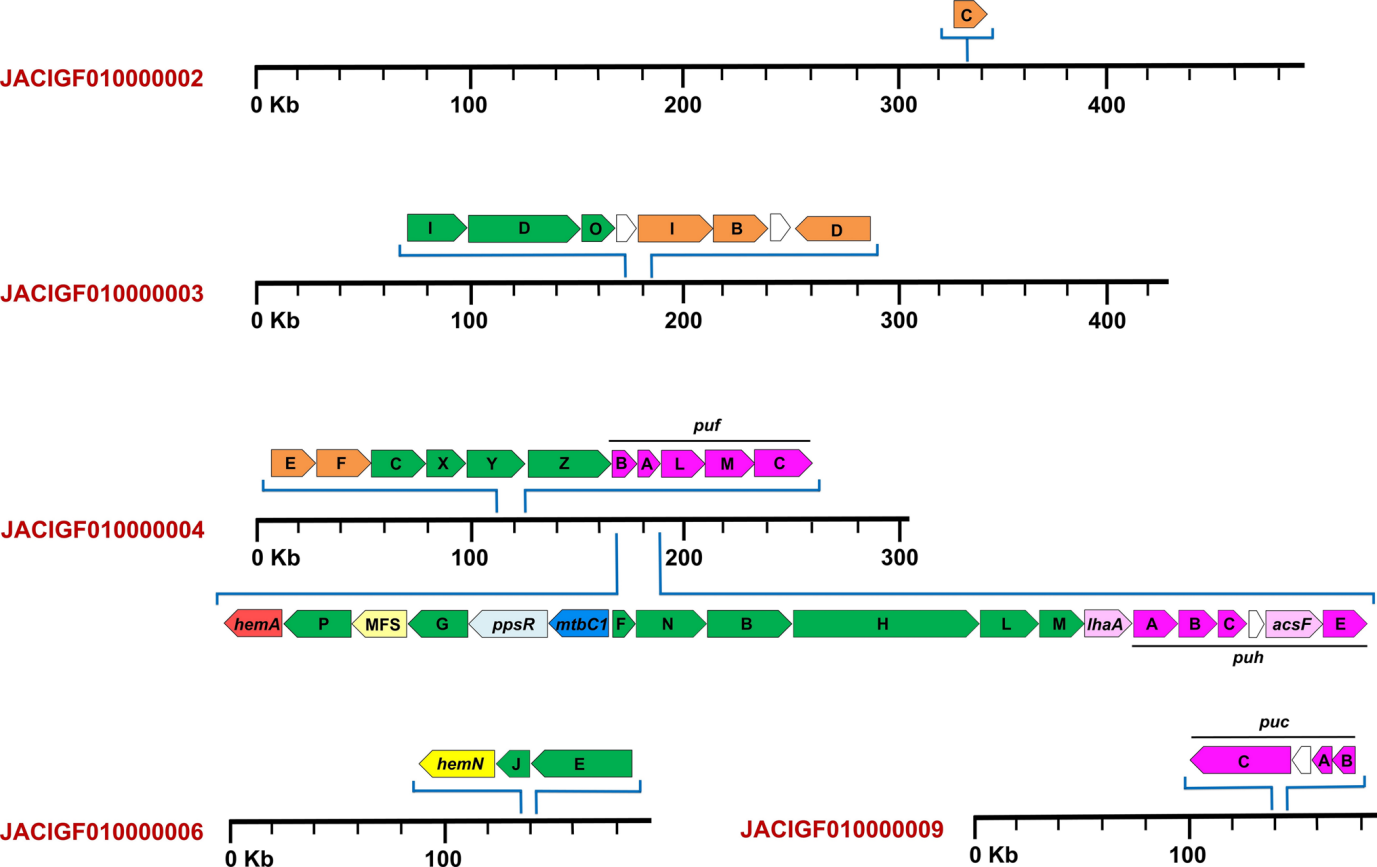
Five of the 24 contigs that make up the *Rts. salexigens* draft genome sequence contain genes encoding photosynthetic functions. Genes encoding bacteriochlorophyll biosynthesis are in green and carotenoid biosynthesis in orange. Genes encoding light-harvesting and reaction center proteins are in purple

Since about half of the *Rts. salexigens* genome appears to have been obtained from horizontal transfers (Fig. 3), it is logical to assume that such may be true of genes encoding photosynthetic functions, as well. However, a compact “photosynthesis gene cluster” (PGC) like that present in PNS bacteria such as *Rhodobacter capsulatus* and *Rhodoferax antarcticus* (Baker et al. 2017), was not evident in the *Rts. salexigens* genome. In *Rba. capsulatus*, for example, genes encoding proteins for the biosynthesis of bacteriochlorophyll and carotenoids and all photocomplex proteins reside in a contiguous superoperon that encompasses about 50 kb (Baker et al. 2017), a fragment of DNA likely obtained by lateral transfer and possibly in a single event. By contrast, genes encoding photosynthetic functions in *Rts. salexigens* are not clustered on the chromosome but instead are dispersed, like what was seen in the genome of the thermophilic purple sulfur bacterium *Thermochromatium tepidum* (Sattley et al. 2022). At least 5 of the 24 contigs of the *Rts. salexigens* draft genome contain genes encoding photosynthetic functions (Fig. 6). More specifically, *bch* genes are found in four distinct “packets” and *crt* genes in three, and *puf*, *puc*, and *puh* genes are separated into different sections of the chromosome (Fig. 6). From these data, we hypothesize that photosynthesis in *Rts. salexigens* originated from several horizontal transfer events followed by random insertion into the organism’s chromosome instead of by acquisition of the entire PGC (or major portions thereof) in a single event. If true, this would be consistent with the overall picture of the *Rts. salexigens* chromosome, a major portion of which apparently has been constructed from multiple gene inputs (Fig. 3).

### Respiration and chemotrophy

*Rts. salexigens* can grow by respiration under fully oxic conditions, and cells were reported to remain significantly pigmented under such conditions (Drews 1981) indicating that oxygen is not as strong a repressor of pigment synthesis in this phototroph as it is in *Rhodobacter* species. From genomic analyses, the *Rts. salexigens* respiratory electron transport chain contains both NADH and succinate dehydrogenase complexes as early components. The former (respiratory Complex I) is encoded by *nuoA–N* while the latter (respiratory Complex II) is a typical FAD-linked succinate dehydrogenase (encoded by *sdhABCD*) that catalyzes the succinate to fumarate oxidation step in the citric acid cycle and likely shows weak bifunctional activity as a fumarate reductase. However, a complete complement of genes encoding an *Escherichia coli*-like NADH-dependent and energy-conserving fumarate reductase (E.C. 1.3.1.6, encoded by *frdABCD*) was absent from the *Rts. salexigens* genome. Homologs showing moderate identity (33–42%) to *E. coli frdAB* were present, but homologs of *frdCD* were absent. Hence, it is unlikely that fumarate respiration can support anaerobic dark growth of *Rts. salexigens*, and such was not reported in the original description of this species (Drews 1981).

Genes encoding both cytochrome *bc*_1_ and cytochrome *bd* complexes were present in the *Rts. salexigens* genome; the former likely participates in photosynthetic electron flow while the latter, encoded by *cytAB*, functions as a terminal oxidase in respiration. However, in addition to cytochrome *bd*, the *Rts. salexigens* genome encodes all four subunits of cytochrome *cbb*_3_ oxidase—a heme-copper oxidase widespread in facultatively aerobic *Pseudomonadota*—along with the accessory protein FixG required for proper assembly of cytochrome *cbb*_3_. Hence, the *Rts. salexigens* genome encodes at least two routes to respiratory O_2_ reduction. Because of the extremely high affinity for O_2_ of the cytochrome *bd* complex, it may function as a terminal oxidase during respiration at low oxygen concentrations. Alternatively, or in addition, it is possible that cytochrome *bd* functions to protect *Rts. salexigens* from sulfide toxicity as it does in *E. coli* (Borisov and Forte 2021; Korshunov et al. 2016). Like cytochrome *bd*, cytochrome *cbb*_3_ oxidases also have high oxygen affinities, and thus which oxidase is expressed during respiration may be controlled by multiple factors. Genes encoding *aa*_*3*_-type cytochrome oxidases, such as those in the PNS bacterium *Rhodobacter sphaeroides* (Daldal et al. 2001), were not encoded in the *Rts. salexigens* genome. However, all genes encoding the components of a bacterial “F-type” ATPase were present.

*Rts. salexigens* uses a limited number of organic compounds as carbon sources to support photoheterotrophic growth; these presumably also function as electron donors for respiratory growth (Drews 1981; Borghese et al. 2001). Acetate, pyruvate, glycerol, glucose, and succinate support good growth of *Rts. salexigens*, as do complex mixtures such as yeast extract and casein (Drews 1981; Rubin and Madigan 1986). Genes encoding the enzymes of central catabolic pathways needed to metabolize these compounds were identified. Genes encoding all enzymes of the glycolytic pathway and all enzymes of the oxidative citric acid cycle were identified in the *Rts. salexigens* genome. In addition, all enzymes of the nonoxidative pentose phosphate pathway were also encoded, including the key proteins transketolase and transaldolase. Likewise, genes encoding homologs of the two key enzymes of the Entner-Doudoroff pathway were identified. Both glycolytic and Entner-Doudoroff enzymes are also encoded in the *Rhodobacter capsulatus* genome, and this organism employs both pathways in the metabolism of fructose but uses only the Entner-Doudoroff pathway to metabolize glucose (Conrad and Schlegel 1977). However, fructose does not support growth of *Rts. salexigens* (Drews 1981), and thus it is possible that both catabolic pathways function during growth on glucose in this phototroph.

Acetate is a particularly good growth substrate for *Rts. salexigens* (Rubin and Madigan 1986); however, genes encoding homologs of isocitrate lyase and malate synthase were not detected in the genome, and thus metabolism of acetate by way of the glyoxylate cycle presumably does not occur. The genome supports a metabolic scenario where acetate is incorporated by a cation-linked (Na^+^ or H^+^) symporter (ActP) and is then converted to acetyl-CoA by acetyl-CoA synthetase or to acetyl-P by acetate kinase, both of which are encoded. Acetyl-CoA carboxylase, which forms the C_3_ intermediate malonyl-CoA, is also encoded, but none of these reactions leads to pyruvate or other key C_3_ intermediates needed for gluconeogenesis. Carboxylation of acetyl-CoA to pyruvate directly by pyruvate synthase also does not occur since a gene encoding a homolog of this enzyme was not identified.

By contrast to these more common routes of acetate metabolism, the formation of C_3_ precursors from acetate in *Rts. salexigens* likely occurs through the activity of enzymes of the ethylmalonyl pathway. This pathway converts crotonyl-CoA (formed from condensation of two molecules of acetoacetyl-CoA) to succinyl-CoA by way of propionyl-CoA. Except for a gene encoding the enzyme hydroxybutanoyl-CoA dehydratase (EC 4.2.1.55), which converts 3-hydroxybutanoyl-CoA to crotonyl-CoA in the ethylmalonyl pathway, all other genes encoding enzymes of this pathway were identified in the *Rts. salexigens* genome. However, this single deficiency may be overcome by the activity of an enoyl-CoA hydratase family protein (EC 4.2.1.17) that is encoded; this enzyme can also form crotonyl-CoA, and as such would allow the ethylmalonyl pathway in *Rts. salexigens* to form essential C_3_ intermediates. The absence of glyoxylate cycle enzymes and the presence of ethylmalonyl pathway enzymes was also found in the highly alkaliphilic but only weakly halophilic PNS bacterium *Roseinatronobacter* (*Rna*.) *bogoriensis* (basonym *Rhodobaca bogoriensis*; Madigan et al. 2023).

Dicarboxylates such as succinate and malate are likely transported into cells of *Rts. salexigens* by the encoded Na^+^/H^+^-linked dicarboxylate symporter that is distinct from the acetate symporter described previously. In addition, two separate TRAP-type C_4_-dicarboxylate transport systems, widespread in diverse gram-negative bacteria (Forward et al. 1997), are also encoded. Genes encoding a specific pyruvate transporter could not be identified, although pyruvate, like acetate, is an excellent growth substrate for *Rts. salexigens* (Rubin and Madigan 1986). It is possible that one of the TRAP-type systems transports pyruvate as reported for the cyanobacterium *Anabaena* (Pernil et al. 2010). Alternatively, pyruvate (and lactate, a substrate also well used by *Rts. salexigens*) may be incorporated by the acetate transporter ActP, mentioned previously, as this has been suggested as a mechanism for pyruvate transport in *Rba. capsulatus* (Borghese et al. 2008) and was also suggested as a pyruvate/lactate transporter in *Rna. bogoriensis* (Madigan et al. 2023). Once incorporated by this (or some unidentified mechanism), pyruvate can enter the citric acid cycle by the activity of pyruvate dehydrogenase or be carboxylated to form malate by an NADP-linked malic enzyme, both of which were encoded. Lactate can be converted to pyruvate by an L-lactate dehydrogenase (LDH), also encoded. However, instead of a yeast-type NADH-linked LDH, the *Rts. salexigens* LDH is annotated as a lactate:ferricytochrome *c*/cytochrome *b*_2_ oxidoreductase (E.C. 1.1.2.3), an enzyme that uses a cytochrome rather than NAD^+^ as oxidant.

Phosphoenolpyruvate generated by glycolysis can also be carboxylated to form oxaloacetate by PEP carboxylase or by the energy-linked enzyme PEP carboxykinase, both of which were also encoded. Thus, at least two anapleurotic CO_2_ fixations are possible in *Rts. salexigens*. Glucose, the only sugar known to support growth of *Rts. salexigens* (Drews 1981), may be transported by a phosphotransferase system (PTS) that was annotated as being specific for mannose and fructose; however, neither of these sugars support growth. This PTS system included a gene encoding enzyme IIA and a HPr phosphocarrier protein, but genes encoding homologs of enzymes IIB and the membrane transporter IIC were not identified. Moreover, genes encoding homologs of other PTS transporters, such as the *ptsG* system widespread in enteric bacteria, or monosaccharide-specific ABC-type transporters, were also not identified in the *Rts. salexigens* genome.

*Rts. salexigens* was reported to be unable to grow photoautotrophically with H_2_, sulfide, or thiosulfate as electron donors (Drews 1981). Curiously, however, the *Rts. salexigens* genome encodes key enzymes of the Calvin cycle: ribulose bisphosphate carboxylase (RuBisCO, both large and small subunits), and phosphoribulokinase. Therefore, the lack of photoautotrophy is presumably not due to an inability to fix CO_2_. Alternatively, this metabolic deficiency may be due to the absence of genes encoding enzymes that oxidize potential electron donors such as H_2_ or reduced sulfur compounds. For example, homologs of genes encoding a “hup”-type uptake hydrogenase (encoded by *hupELSTUV* in *Rhodobacter sphaeroides* and several other PNS bacteria capable of autotrophic growth with H_2_ as electron donor) were absent from the *Rts. salexigens* genome. Likewise, genes encoding thiosulfate oxidation (the *sox* operon) were absent as were genes encoding enzymes catalyzing sulfide oxidation, such as the sulfide:flavocytochrome *c* and sulfide:quinone oxidoreductases of *Allochromatium vinosum* (Weissgerber et al. 2011). Thus, the presence of genes encoding Calvin cycle enzymes in *Rts. salexigens* means that photoautotrophy, if such is possible, is supported by an atypical electron donor. Alternatively, it is possible that Calvin cycle enzymes are expressed in *Rts. salexigens* only under certain nonautotrophic growth conditions. Such has been observed in PNS bacteria grown photoheterotrophically on highly reduced organic compounds such as butyrate. Under such conditions, Calvin-cycle-driven CO_2_ fixation functions as an electron sink to consume excess NADH generated during catabolism and integration of the organic substrate into new cell material (McKinlay and Harwood 2010).

*Rts. salexigens* likely stores carbon as the intracellular polymer polyhydroxyalkanoate (PHA). Acetate should feed into PHA when it is present in excess because genes encoding the enzymes acetyl-CoA acetyltransferase (beta-acetoacetyl coenzyme A thiolase, PhaA), acetoacetyl-CoA reductase (PhaB), and PHA synthase (PhaC) were all identified in the genome. When PHA carbon is needed, depolymerization can proceed by the activity of PHA depolymerase encoded by *phaZ*. In addition to carbon, *Rts. salexigens* should be able to store phosphate as insoluble polyphosphate (PP) because two PP kinase genes were identified in the genome. The first encoded a PP kinase of similar size and of moderate homology to the large PP kinase of *E. coli* (PP kinase 1), while the second encoded PP kinase was of similar size and of moderate homology to *E. coli* PP kinase 2. In *E. coli* both enzymes can polymerize phosphate from nucleoside triphosphates; however, PP kinase 1 preferentially synthesizes polyphosphate while PP kinase 2 preferentially consumes phosphate from PP (Neville et al. 2022).

### Nitrogen and sulfur metabolisms

*Rts. salexigens* has a curious history concerning nitrogen metabolism. In the species description, the organism was said to be a glutamate auxotroph; that is, both phototrophic and chemotrophic growth required glutamate as an N source (Drews 1981). The fact that ammonia did not support growth of *Rts. salexigens* seemed highly unusual, and hence this conclusion was revisited in the work of Rubin and Madigan (1986). The latter found that *Rts. salexigens* grows optimally near pH 7 but only poorly at alkaline pH. Moreover, it was discovered that when *Rts. salexigens* was grown photoheterotrophically on acetate with ammonium as sole N source, only scant growth occurred while the pH of the medium rose to near pH 8, presumably from excretion by the inoculum of some highly basic substance. However, if pH was controlled by adding pyruvate or by the addition of organic buffers such as MOPS, luxurious growth on ammonium as N source occurred. If both ammonium and glutamate were present in the medium for *Rts. salexigens*, glutamate was used to the exclusion of ammonium, indicating that glutamate was indeed the preferred N source (Rubin and Madigan 1986). Subsequent growth experiments showed that in addition to ammonium, several other amino acids, as well as complex N sources such as casamino acids or yeast extract, also supported good growth of *Rts. salexigens*. By contrast, no growth of *Rts. salexigens* occurred with nitrate or urea as N sources (Rubin and Madigan 1986).

Growth of *Rts. salexigens* on aspartate, glutamate or glutamine as N sources was accompanied by significant H_2_ production, presumably due to nitrogenase depression, and was an early clue that the organism could fix dinitrogen. Subsequent growth tests using N_2_ as sole N source supported by strong acetylene reduction in N_2_- or glutamate-grown cells confirmed this (Rubin and Madigan 1986). The *Rts. salexigens* genome encodes a Mo-containing nitrogenase and dinitrogenase reductase (*nifHDK*) and also contains the genes *nifENB*, which encode proteins of nitrogenase cofactor biosynthesis. These two gene sets are the minimum required for an organism to be diazotrophic (Dos Santos et al. 2012). However, in addition to these, the *Rts. salexigens* genome encodes homologs of NifA, a transcriptional activating protein; NifWXZ, nitrogenase maturation and assembly proteins; NifT, a putative nitrogen fixation regulatory protein; and NifV, homocitrate synthase. No genomic evidence was obtained for vanadium or iron-only nitrogenases (Harwood 2020) in *Rts. salexigens*.

Ammonium is incorporated into cells of *Rts. salexigens* by a dedicated transporter of the AmtB type, common in marine bacteria. Once in the cytoplasm, ammonium can be converted to organic form in *Rts. salexigens* in at least two ways. First, the amino acid alanine can be formed by an NADH/NADPH-linked alanine dehydrogenase; activity of this enzyme was detected at high levels in extracts of ammonia-grown cells (Rubin and Madigan 1986). By contrast, although a classical NAD-linked glutamate dehydrogenase (E.C. 1.4.1 2) is encoded in the *Rts. salexigens* genome, its activity was not detected in extracts of cells grown on either glutamate or ammonium as N source (Rubin and Madigan 1986). However, glutamine synthetase is encoded, and so once glutamate is in the cell, it can be aminated to form glutamine. Transamination reactions using glutamine as N-donor can then form other organic N compounds. Genes encoding both large and small subunits of an NADPH-linked glutamate synthase, an enzyme that converts two glutamates into oxoglutarate and glutamine, were also identified in the *Rts. salexigens* genome.

Although glutamate is an excellent N source for *Rts. salexigens* (Drews 1981; Rubin and Madigan 1986), exactly how this phototroph incorporates glutamate is unclear. Genes encoding a specific glutamate transporter analogous to the polar amino acid transporters were not found. However, at least two alternative possibilities for glutamate transport were identified. The *Rts. salexigens* genome encodes a sodium/proton-linked dicarboxylate symporter with significant homology to GltP, a proton-linked glutamate symporter in *E. coli* and several other bacteria (Slotboom et al. 1999); GltP can also transport aspartate, another excellent N source for *Rts. salexigens* (Rubin and Madigan 1986). In addition, a sodium-linked alanine:glycine transporter is encoded that possibly transports glutamate and other amino acids as well.

Neither nitrate, nitrite, nor urea is used by *Rts. salexigens* as a N source (Rubin and Madigan 1986). As expected, no genes encoding a nitrate transporter—typically an ABC-type in most nitrate-assimilating bacteria—could be identified, and no urea transporter was identified, either. Moreover, no genes encoding enzymes for dissimilatory nitrate reduction were found, indicating that dark anaerobic growth by respiration of nitrate to either ammonia or N_2_ (denitrification) does not occur in *Rts. salexigens*. If fumarate respiration is also impossible (see earlier discussion of succinate dehydrogenase), the only chemotrophic metabolism possible in *Rts. salexigens* would be aerobic respiration. Glucose is used as a carbon source for photoheterotrophic growth (Drews 1981), but whether glucose supports respiratory or fermentative growth is unknown.

*Rts. salexigens* can grow in defined culture media containing only sulfate as a biosynthetic sulfur source (Drews 1981; Rubin and Madigan 1986). Sulfate acquisition by *Rts. salexigens* is facilitated by an ABC-type transporter encoded by the genes *sbpP* and *cysUWA* and then converted to adenosine 5′-phosphosulfate (APS) and 3′-phosphoadenosine 5′-phosphosulfate (PAPS) by CysNDC. PAPS is then reduced to sulfite by a thioredoxin-linked PAPS reductase (CysH) and further reduced by an NADPH:FAD-linked sulfite reductase (encoded by *cysJI*) to sulfide for biosynthesis of cysteine, methionine, and other S-containing intermediates. Genes encoding a direct transporter of sulfide or sulfite into cells of *Rts. salexigens* could not be identified, consistent with the fact that sulfide does not support autotrophic growth despite the organism’s genome encoding Calvin cycle enzymes.

### Phospholipids

**Fig. 7:**
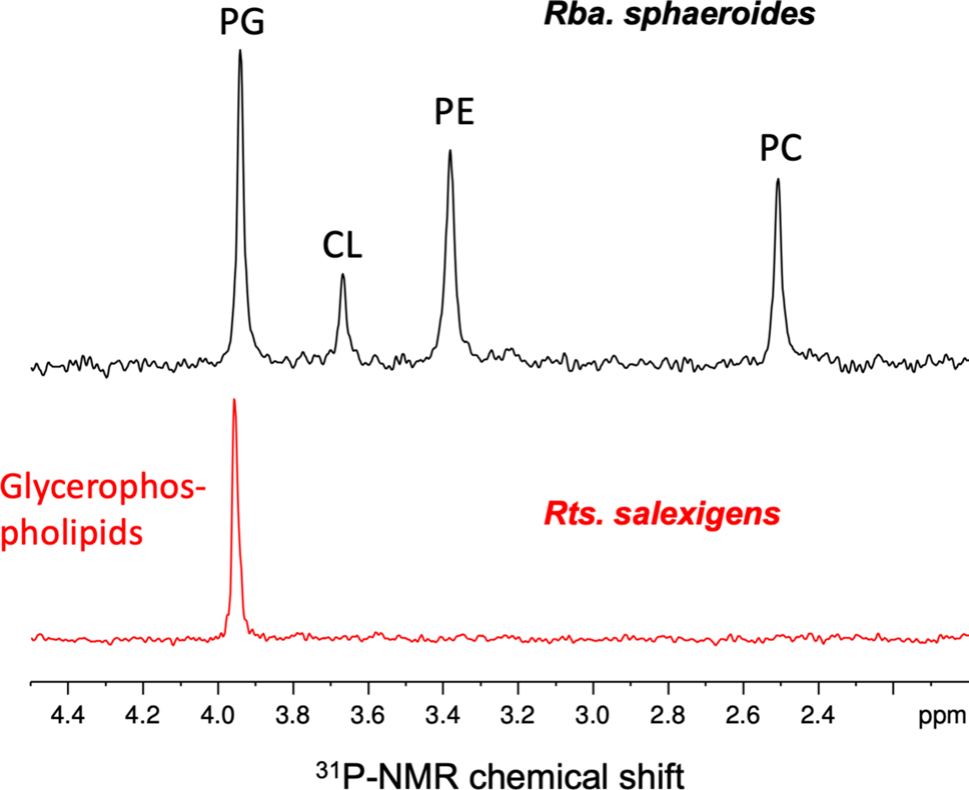
^31^P-NMR spectra of phospholipids extracted from ICMs of *Rts. salexigens* compared with those from *Rhodobacter sphaeroides*. Abbreviations: PG, phosphatidylglycerol; CL, cardiolipin; PE, phosphatidylethanolamine; PC, phosphatidylcholine. In *Rts. salexigens*, several glycerophospholipids are present (see text) that overlap with the ^31^P signal for PG

In analyses of the genomics supporting phospholipid biosynthesis in *Rts. salexigens*, it was astonishing to find that the organism lacked genes necessary to produce many of the major phospholipids commonly found in bacterial membranes (Sohlenkamp and Geiger 2016); genes encoding phosphatidylglycerol (PG) biosynthesis were the only ones identified. Biochemical confirmation of this surprising finding was achieved through ^31^P-NMR analysis of *Rts. salexigens* ICMs that clearly showed them to contain almost exclusively glycerophospholipids. By contrast, ICMs from *Rhodobacter sphaeroides*, used as a control, contained PG as well as phosphatidylethanolamine (PE), phosphatidylcholine (PC, also called lecithin), and cardiolipin (CL) (Fig. 7). Phosphatidylinositol (PI) and phosphatidylserine (PS) are absent from ICMs of both of these species.

The ^31^P-NMR signal in Fig. 7 contained several glycerophospholipids including PG and other diacylglycerophosphoglycerols. *Rts. salexigens* contains most of the genes encoding enzymes that convert glycerol-3-P into PG; these include genes encoding acylglycerol-3P acyltransferase (E.C. 2.3.1.51), phosphatidate cytidylytransferase (E.C. 2.7.7.41, which generates the key phospholipid intermediate CDP-diacylgycerol), and phosphatidylgycerol-phosphatase (E.C. 3.1.3.27). A gene encoding a homolog of CDP-diacylgycerol-glycerol-3 phosphate-phosphatidyltransferase (E.C. 2.7.8.5)—the terminal step in phosphatidylgycerol biosynthesis—could not be identified. However, a gene encoding a protein that showed 39% identity to this enzyme from *Escherichia coli* was identified and was co-annotated in the IMG database as both a CDP-diacylgycerol-glycerol-3-phosphate-phosphatidyltransferase and a cardiolipin synthase (E.C. 2.7.8.41). Since cardiolipin was not detected in ICMs of *Rts. salexigens* (Fig. 7), it is likely that this enzyme functions to catalyze the terminal step in PG biosynthesis. A gene encoding a specific E.C. 2.7.8.5 activity is also absent from the genomes of both *Rhodovibrio* species (Table 1), the PNS bacteria *Rhodobacter sphaeroides*, *Roseinatronobacter bogoriensis*, and *Rhodospirillum rubrum* (Satoh et al. 2023), and a host of other *Alphaproteobacteria* all of which contain phosphatidylglycerol in their membranes (Sohlenkamp and Geiger 2016). Thus, the absence of a dedicated CDP-diacylgycerol-glycerol-3 phosphate-phosphatidyltransferase in *Rts. salexigens* is not unusual and is obviously not required to produce phosphatidylglycerol or the other glycerophospholipids observed in this phototroph.

CDP-diacyl-glycerol is a major hub in the biosynthesis of several phospholipids (Sohlenkamp and Geiger 2016). In the *Rts. salexigens* genome, proteins are encoded that allow this hub to feed into PG biosynthesis but not into the biosynthesis of PS, PE, PI, or PC. No homolog of the gene encoding CDP-diacyl-glycerol serine *O*-phosphatidyltransferase was present, indicating that the enzyme necessary to produce the precursor of PE and PC was absent. Additional missing genes include one encoding PS carboxylase (E.C. 4.1.1.65), which produces PE by decarboxylation of PS, and a gene encoding PC synthase (E.C. 2.7.8.24), which produces PC directly from CDP-diacyl-glycerol and choline. Thus, the absence of these common bacterial phospholipids from the ICM of *Rts. salexigens* (Fig. 7) is in full agreement with its minimal complement of genes encoding phospholipid biosyntheses.

As previously mentioned, cardiolipin was undetectable in ICMs from *Rts. salexigens* (Fig. 7), although a protein is encoded that annotates as cardiolipin synthase. However, this enzyme likely functions instead in the conversion CDP-diacyl-glycerol to phosphatidylglycerol-phosphate. Authentic cardiolipin synthase (E.C. 2.8.7.41) from *Escherichia coli* is a much larger protein than the putative *Rts. salexigens* cardiolipin synthase and showed no detectable homology to this or any proteins from *Rts. salexigens*. Thus, along with other major phospholipids, the absence of cardiolipin from ICM of *Rts. salexigens* is likely due to the lack of a gene encoding a dedicated cardiolipin synthase.

This limited repertoire of phospholipids in *Rts. salexigens* ICMs may also extend to the other two phototrophs listed in Table 1. Preliminary examinations of the genomes of *Rhodovibrio salinarum* (JGI/IMG genome ID 8062844039) and *Rhodovibrio sodomensis* (JGI/IMG genome ID 2993873675) indicate that although genes encoding PG biosynthesis are clearly present in both species, genes encoding enzymes for the production of PS, PE, and PI are absent. However, the genetic blueprint of *Rhv. sodomensis* (but not *Rhv. salinarum*) shows that PC biosynthesis should be possible from diacylglycerol. In addition, although absent from the *Rts. salexigens* genome, genes encoding proteins that convert PE to PC are present in both *Rhodovibrio* species but how PE would be made in these organisms is unclear. *Rts. salexigens* and the two *Rhodovibrio* species differ in one other way. The genomes of both *Rhodovibrio* species contain homologs of the genes *clsAB* that encode authentic cardiolipin synthase, the enzyme that forms cardiolipin from two molecules of PG, and thus they likely produce CL. Homologs of *clsAB* were not identified in the *Rts. salexigens* genome.

### Sphingolipids

In addition to glycerolphospholipids, a small but significant fraction of the total membrane lipids of *Rts. salexigens* consisted of the amide-containing lipids called sphingolipids. Although ornithine- and glutamine-containing aminolipids have been reported from aerobic purple bacteria (Smith et al. 2021) and an aminoglycosphingolipid from the green sulfur bacterium *Chlorobium limicola* (Jensen et al. 1991), the occurrence of sphingolipids in *Rts. salexigens* is the first report of such lipids in purple phototrophic bacteria. Sphingolipids, present in eukaryotic cell membranes, are rarely found in bacteria but are common among species of gram-negative bacteria of the genus *Sphingomonas* and its relatives (*Alphaproteobacteria*) (Balkwill et al. 2006).

The sphingolipids in *Rts. salexigens* were not glycosylated or phosphorylated as they typically are in *Sphingomonas* species but instead were in the form of dihydroceramides (dihydrosphingosine plus a C_14_ or C_16_ fatty acid connected to the dihydrosphingosine by an amide bond). A gene encoding serine palmitoyltransferase, the enzyme that catalyzes the first step in sphingosine biosynthesis, was identified in the *Rts. salexigens* genome, along with a second enzyme annotated as a sphingolipid/fatty acid hydroxylase that may have multiple functions as a hydroxylase. Genes encoding sphingolipid biosynthesis in *Rts. salexigens* likely originated from horizontal transfers from donors in the *Sphingomonadales*, as genes from this bacterial order were detected in the *Rts. salexigens* genome (Fig. 3).

### The *Rts. salexigens* cell envelope

The cell envelope of cells of *Rts. salexigens* consists of a particle-studded surface layer overlying a thin outer membrane (Golecki and Drews 1980). This structure is almost exclusively composed of protein, and carbohydrates are barely detectable; the single form of protein present contains a large excess of acidic over basic amino acids, a pattern common in proteins that are in contact with high salinities (Tadros et al. 1982; Evers et al. 1984). Although tests for the presence of peptidoglycan in *Rts. salexigens* were negative and no murein sacculus was detectable by electron microscopy, cells treated with EDTA/lysozyme were shown to form spheroplasts (Golecki and Drews 1980). In a later biochemical study, cells of *Rts. salexigens* were shown to contain small amounts of peptidoglycan including the signature amino sugars glucosamine and muramic acid and the peptidoglycan cross-linking amino acid diaminopimelic acid along with large amounts of protein (Tadros et al. 1982). The latter supports the prediction of Golecki and Drews (1980) that protein is the main if not sole component of the *Rts. salexigens* cell envelope.

Genomic evidence for the presence of peptidoglycan in *Rts. salexigens* was obtained from the identification of genes encoding homologs of two murein lytic transglycosylases, MltA and MltB, the peptidoglycan biosynthesis protein MurJ, glucosamine-1-P *N*-acetyltransferase (that forms *N*-acetylglucosamine), and *N*-acetylmuramate-P uridylyltransferase (that forms *N*-acetylmuramic acid). Also present were genes encoding homologs of MurA–F and MurYG that collectively lead to the large peptidoglycan precursor UDP-muramate-*N*-acetyl Ala-D-Glu-DAP-D-Ala-D-Ala. Genes missing included MurMN and FemABX (which substitute lysine for DAP), consistent with the biochemical finding that *Rts. salexigens* peptidoglycan is of the “DAP type” (Tadros et al. 1982).

It is curious to consider whether there is any connection between the morphology of *Rts. salexigens* ICM and the fact that glycerophospholipids constitute virtually all of the membrane lipids. In thin-sectioned electron micrographs, the ICMs of *Rts. salexigens* lie parallel to the long axis of the cell (Golecki and Drews 1980) in a manner reminiscent of the ICM architecture of the PNS bacteria *Rhodomicrobium vannielii* and *Rhodopseudomonas palustris* (Drews 1978); in cross section, *Rts. salexigens* ICMs appear as concentric circles (Drews 1981). The outer membrane (OM) of *Rts. salexigens* is quite thin (Golecki and Drews 1981) and presumably contains only PG as a phospholipid component. The *Rts. salexigens* OM also lacks lipopolysaccharide (Tadros et al. 1982). Despite this atypical OM chemistry, several widely distributed OM-located proteins are encoded in the organism’s genome. For example, two genes encoding the OM efflux protein TolC were identified, along with genes encoding BamADE (the minimal functional form of the β-barrel assembly machinery proteins that assist in assembling OM proteins, Wang et al. 2024), at least one DCaP family porin protein, and proteins encoding lipoprotein transport and OM transporters. A noncovalently bound peptidoglycan-associated lipoprotein related to the Pal proteins that are widespread in gram-negative bacteria is also encoded in the *Rts. salexigens* genome; Pal typically functions to stabilize the OM at the cell restriction site during cell division (Szczepaniak et al. 2020). An OmpA protein with significant homology to that from a host of gram-negative bacteria was also encoded. Among its many functions, OmpA helps anchor the OM to the peptidoglycan layer and thereby confer cell envelope stability. A homolog of the Braun protein, a small covalently bound lipoprotein that tethers the OM to peptidoglycan in *E. coli* and several other gram-negative bacteria (Bahadur et al. 2021; Sheng et al. 2023), was not encoded in the *Rts. salexigens* genome. Thus, despite being topologically quite thin and lacking lipopolysaccharide, an OM that in most other respects resembles that of other gram-negative bacteria is present in *Rts. salexigens*.

### Hopanoids in *Rts. salexigens*

One or more of the outer membrane, cytoplasmic membrane, and intracytoplasmic membranes of cells of *Rts. salexigens* are likely strengthened by hopanoids (bacteriohopanepolyols, BHPs). These sterol-like lipids are widely distributed in bacteria where they are thought to help the organism withstand environmental stressors such as extremes of pH or salinity (Belin et al. 2018; Garby et al. 2017). BHPs have been detected in a few species of phototrophic purple bacteria including *Rts. salexigens* (Jahnke et al. 2014; Mayer et al. 2021), and genes encoding homologs of some hopanoid biosynthetic proteins were identified in the *Rts. salexigens* genome. These included HpnC (squalene synthase), which forms squalene (C_30_) from two molecules of the C_15_ compound farnesyl pyrophosphate, and a gene encoding squalene-hopene/tetraprenyl-beta-curcumene cyclase (E.C. 5.4.99.17) that showed 36% identity to the more widely distributed squalene cyclase. Both enzymes form the hopanoid diploptene by cyclizing squalene. Also encoded in the *Rts. salexigens* genome was the enzyme HpnH, which adds an adenosyl group to diploptene to form adenosylhopane, and HpnB, a glycosyltransferase that catalyzes the addition of carbohydrates to form glycosylated hopanols; the genes *hpnB* and *hpnC* lie adjacent to one another in the same *Rts. salexigens* operon. Absent from the *Rts. salexigens* genome were genes encoding homologs of HpnG, which cleaves adenine from adenosylhopane, and HpnP, which methylates hopanol at the C-2 position; both function in the biosynthesis of modified hopanoids in the purple bacterium *Rhodopseudomonas palustris* (Mayer et al. 2021). Although encoded, it is unclear whether HpnB can function in *Rts. salexigens* without HpnG activity because the latter leads to the production of the extended hopanoid bacteriohopanetetrol, which is the substrate for HpnB to form glycosylated hopanoids (Belin et al. 2018).

In a study of lipids from a gypsum-encrusted microbial mat and from pure cultures of purple bacteria thought to inhabit the mat (Jahnke et al. (2014), *Rts. salexigens* was one of several species of marine PNS bacteria in which BHPs were detected. Of a total of 65 μg BHP/g cell dry weight detected in *Rts. salexigens*, major amounts of a C_32_ BHP were present along with minor amounts of C_30_, C_31_, and 3-methyl BHPs. Synthesis of the latter should require the activity of the enzyme HpnR that methylates hopanol at the C-3-position, and among purple bacteria, 3-mBHPs have only been detected in the acidophilic PNS bacterium *Rhodopila globiformis* (Mayer et al. 2021). Nevertheless, a BLAST analysis of the *Rpi. globiformis* HpnR sequence yielded a hit to a *Rts. salexigens* protein showing relatively low (26%) sequence identity to HpnR but yielding a high bit score and low e score. The protein was annotated as an anaerobic magnesium-protoporphyrin IX monomethyl ester cyclase (BchE), a radical SAM enzyme that normally functions in bacteriochlorophyll biosynthesis. This protein contains a B_12_ binding site and belongs to a protein superfamily that includes methyltransferases, so it is possible that it is a bifunctional protein and also methylates *Rts. salexigens* C_32_ BHP. In sum, *Rts. salexigens* is genetically equipped to produce a variety of BHPs, and these lipids may strengthen the membranes of this phototroph to withstand osmotic stress from the variations in salinity it experiences in its habitat.

### Osmotic protection

To maintain positive osmotic pressure in saline environments, halophilic bacteria must either synthesize or accumulate solutes in their cytoplasm, and in this connection, the organic compounds betaine and ectoine are the major compatible solutes found in halophilic purple bacteria (Imhoff et al. 2020). Ectoine is widespread among marine species of *Rhodobacteraceae* but is not synthesized by* Rts. salexigens* (Imhoff et al. 2020). Instead, this moderate halophile maintains osmotic balance by accumulating and/or synthesizing a single osmoprotectant, glycine betaine.

The habitat of *Rts. salexigens*—evaporating seawater containing decaying seaweeds (Drews 1981)—is undoubtedly quite organic-rich and should contain glycine betaine leaked from decomposing vegetation (Giri 2011; Imhoff et al. 2020). The *Rts. salexigens* genome contains three copies of *betT*, which encode a multi-substrate transporter that incorporates betaines of glycine, choline, or proline. Because of the widespread salinity tolerance of *Rts. salexigens* (growth from 4–20% NaCl), it is tempting to speculate that expression of *betT* may be regulated in this organism by salinity with perhaps one copy expressed at low salinity and others expressed as salinity increases. This would be consistent with a hypothesis that the moderate salinity optimum observed for *Rts. salexigens* (about 7% NaCl) has evolved as it has because it is closest to what the organism typically experiences in its marine evaporation pond habitat but that highly variable salinities are temporarily possible. Such evaporation ponds are flushed with seawater periodically, and as drying occurs, hypersaline conditions gradually increase. Thus, mechanisms to deal with both low and high salinities are necessary. The *Rts. salexigens* genome also encodes a homolog of OpuB, a high-affinity ABC-type choline transporter, and OpuA, a high-affinity ABC-type glycine betaine transporter. Thus, *Rts. salexigens* seems well equipped to incorporate glycine betaine from its habitat whether it is present in abundance or is limiting.

If osmotic solutes are unavailable, such as when *Rts. salexigens* is grown in laboratory culture, the organism must biosynthesize them. If choline is incorporated, it is likely a dead-end osmotic substrate. In contrast to *Rhodovibrio* species (Table 1), choline cannot be oxidized to glycine betaine in *Rts. salexigens* because the genome lacks *betBA*, which encode betaine-aldehyde dehydrogenase and choline dehydrogenase, respectively, enzymes needed for this transformation (Gregory and Fidelma Boyd 2021). Also absent are genes encoding homologs of the proteins GbsAB, wide-specificity aldehyde dehydrogenases that biosynthesize glycine betaine from choline in *Bacillus* species (Rath et al. 2020). Likewise, choline cannot be converted to trimethylamine in *Rts. salexigens* because it lacks a gene encoding choline:trimethylamine lyase, an enzyme found in sulfate-reducing and some other obligately anaerobic bacteria (Craciun and Balskus 2012). Thus, glycine, either accumulated from the environment or biosynthesized from central metabolic processes (all genes necessary for glycine biosynthesis were identified) is the likely starting substrate for glycine betaine biosynthesis in *Rts. salexigens*. Its genome contains homologs of the two enzymes necessary to do this—glycine sarcosine methyltransferase (GMT) and dimethylglycine methyltransferase (DMT). The former protein, annotated in the *Rts. salexigens* genome as a SAM-dependent methyltransferase, had 63% amino acid identity to GMT from the extremely halophilic purple bacterium *Halorhodospira*. The second protein, annotated as sarcosine/dimethylglycine N-methyltransferase, showed 49% amino acid identity to the *Halorhodospira* homolog and 50% identity to the protein from *Marichromatium purpuratum* (Imhoff and Trüper 1980), a marine species of purple bacteria that grows optimally at 5% NaCl, close to that of *Rts. salexigens.* It is also possible that the *Rts. salexigens* DMT homolog catalyzes both methylations of glycine, as such dual enzymatic activity is common in the DMT from many halophiles (Imhoff et al. 2020).

The genetics of osmotic protection in *Rts. salexigens* is less extensive than in its *Rhodovibrio* phylogenetic neighbors (Fig. 1 and Table 1). In the Dead Sea halophile *Rhv. sodomensis* and the solar saltern halophile *Rhv. salinarum*, besides incorporating betaines from the *betT*-encoded uptake system, these species can biosynthesize glycine betaine from choline and transport proline (through the activities of ProVWX). Moreover, these two halophiles can also biosynthesize the osmolyte ectoine from aspartate through the activities of the proteins EctABC (Imhoff et al. 2021), proteins not encoded in the *Rts. salexigens* genome.

### Motility and chemotaxis

**Fig. 8:**
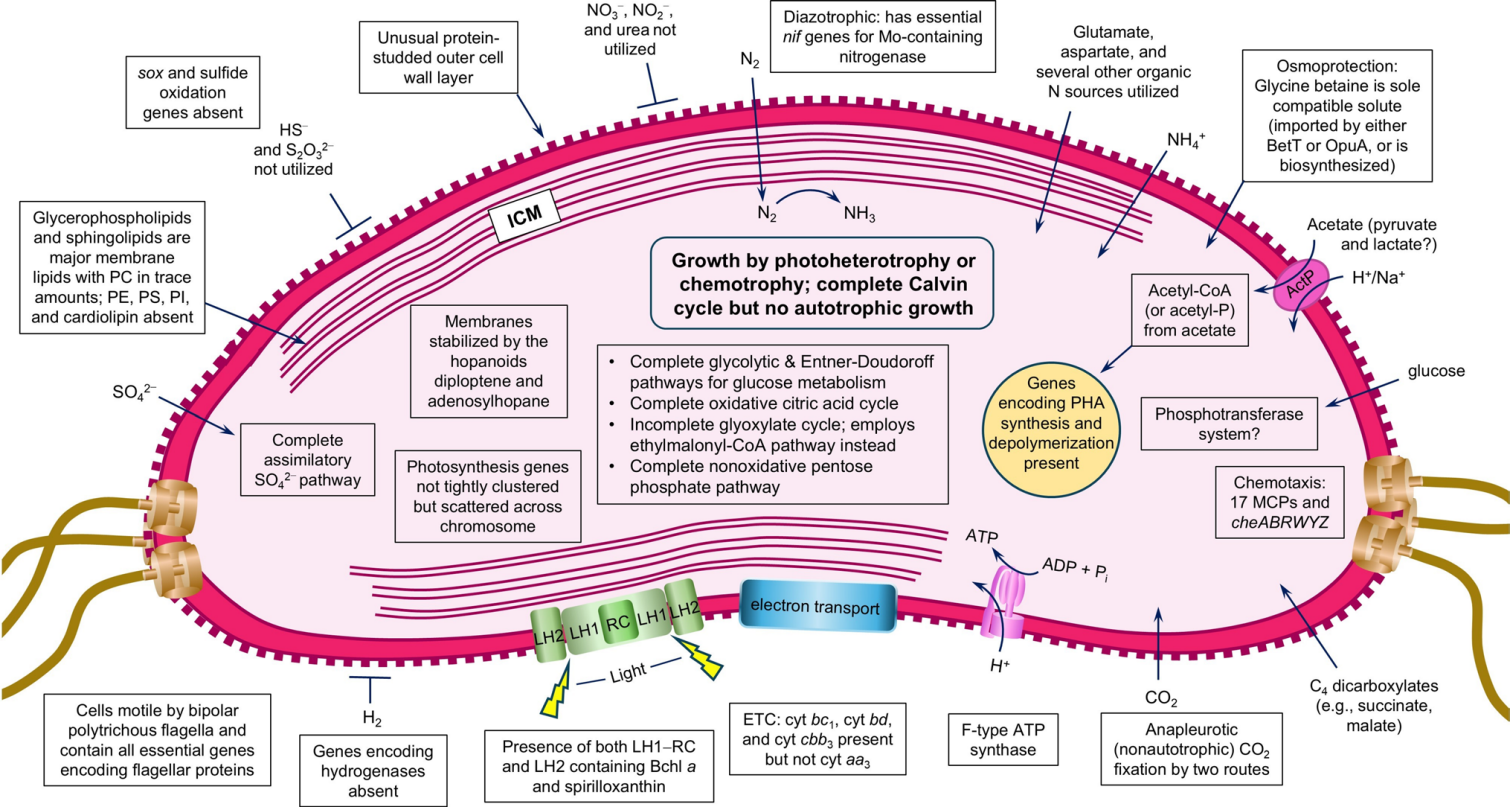
Overview of the genomics and biochemistry of *Rts. salexigens*

*Rts. salexigens* cells are motile by the activity of bipolar polytrichous flagella (Drews 1981) (Fig. 8). The organism’s genome showed all genes encoding the flagellar MS/C ring except for FliHJ. All flagellar rod, P/L ring, and hook genes (*flgA–L*) were identified as were two of the genes (*flhAB)* encoding a Type-3 secretion system. Although a gene encoding the flagellar filament protein (FliC) was present, homologs of FliDST, which encode the filament cap and chaperone, were not. Moreover, the essential stator proteins MotAB were encoded while optional stator proteins (MotCD) were not. However, in a final analysis, all essential genes encoding flagellar proteins (*fliCFGIPQR*, *flgBCDEEF*, and *flhAB*) (Liu and Ochman 2007) were present in the *Rts. salexigens* genome. None of the missing flagellar genes are essential for motility and are often absent from the genomes of other motile *Alphaproteobacteria* (Liu and Ochman 2007), the phylum to which *Rts. salexigens* belongs (Fig. 3).

The *Rts. salexigens* genome has an extensive suite of genes encoding chemotaxis functions. Seventeen genes encoding methyl-accepting chemotaxis proteins (MCP) of lengths from 430 to 733 amino acids were identified that showed 30–45% amino acid identity to one another. Considering the organic-rich habitat of *Rts. salexigens*—decaying plant material—an extensive array of chemoreceptors sensitive to a wide variety of organic compounds would likely be of significant advantage. The *che* genes *cheABRWYZ* are present in the *Rts. salexigens* genome but *cheCDX* are missing. CheC and CheX are proteins that modulate the activity of CheY, the flagellar switch, but the presence of the phosphatase CheZ in *Rts. salexigens* takes on this function, as it does in *Escherichia coli* and many chemotactic gram-negative bacteria. By contrast, the proteins CheCD are found in *Bacillus* species, *Thermotoga*, and a few other bacteria, and carry out a similar function in ultimately affecting the activity of the flagella “switch”, CheY.

## Summary and conclusions

Figure 8 provides a pictorial overview of the biology of *Rts. salexigens* based on our genomic analyses and the handful of previously published papers that have examined structural and biochemical features of this unusual PNS bacterium. Since roughly half of the genome of *Rts. salexigens* is composed of genes unique to the order *Rhodothalassiales* (Fig. 3), and keeping in mind that *Rts. salexigens* is the only known species in this order, there are undoubtedly other highly unusual features of this organism that have not been revealed here or in previous studies of this phototroph. Nevertheless, our genomic analysis of *Rts. salexigens* has confirmed several important features of this halophilic phototroph and exposed some new aspects of its physiology. Some high points include (1) a highly chimeric genome in which almost half of the genes in this phototroph have phylogenetic origins in families of *Alphaproteobacteria* other than *Rhodothalassiaceae*; (2) the presence of genes encoding Calvin cycle proteins but the absence of autotrophy due to an inability to oxidize common reduced electron donors; (3) the scattering of photosynthesis genes across the genome; (4) redundant systems for incorporating or biosynthesizing glycine betaine, the sole osmolyte; (5) confirmation of the unusual pattern of N metabolism; and (6) a unique phospholipid profile and the genetic capability to synthesize hopanoids.

Although it is likely that the capacity for phototrophy in *Rts. salexigens* is the result of horizontal gene transfer(s), the phylogenetic roots of this phototroph are quite distant from the clusters of highly related PNS bacteria in the families *Rhodospirillaceae* or *Rhodobacteraceae*, and thus *Rts. salexigens* stands out as an excellent candidate for the further study of photosynthesis. In this regard, it will be insightful to study photocomplexes from *Rts. salexigens* to see whether this phototroph’s unusual phospholipid composition and ICM architecture helps stabilize photosynthetic processes in ever-shifting osmotic environments. In this connection, basic studies of photosynthesis in this easily cultured PNS bacterium could benefit applied studies of plant saline stress and provide new insights on how sunlight is harvested in extreme environments.
